# Comments on “Knowledge Translation of the PERC Rule for Suspected Pulmonary Embolism: A Blueprint for Reducing the Number of CT Pulmonary Angiograms”

**DOI:** 10.5811/westjem.2018.2.36889

**Published:** 2018-03-13

**Authors:** Jeffrey Dubin, Matthew Wilson, William Frohna

**Affiliations:** MedStar Emergency Physicians, MedStar Washington Hospital Center/Georgetown Department of Emergency Medicine, Washington, District of Columbia

To the Editor:

We read with enthusiasm the recent publication of Drescher et al. and applaud their department’s commitment to embedding evidence-based medicine (EBM) for best practice in the diagnosis of pulmonary embolism (PE) in their culture through education and computerized decision support (CDS)1. Our healthcare system had similar findings with implementing a CDS tool for our emergency departments (ED), which utilizes the revised Geneva criteria for risk stratification as opposed to Well’s criteria.

We designed a CDS tool using the revised Geneva criteria to first risk stratify patients with suspected PE to low, moderate, or high risk. The tool next directed providers to use the Pulmonary Embolism Rule-out Criteria (PERC) for low risk, and if not PERC negative or if moderate risk, order d-dimer testing. The tool indicates that high-risk patients and those with positive d-dimer are appropriate for computed tomography pulmonary angiograms (CTA). The tool was inserted in the electronic medical record (EMR) at six EDs in a single healthcare system using the same EMR (Cerner Corporation, North Kansas City, Missouri.) After obtaining IRB approval, we studied the effect of the EMR CDS tool. We hypothesized that post-implementation the number of CTAs performed would decrease and the diagnostic yield would increase.

Total CTA utilization proportionally decreased post implementation with 4,981 CTAs of 311,313 (1.6%) visits in 2014 compared to 4,608 CTA of 307,200 (1.5%) for 2015, p =0.001. The proportion of patients with a positive study of all those who had CTA was not significantly different from 2014 to 2015 (5.7% vs. 6.6%, p=0.68). In the post-implementation group, the percent positive CTA was 6.7% when the EMR tool was used (263 positive of 3,926) but not significant in comparison to when it wasn’t used [5.7% (39 positive of 682), p=0.34].

Although our study suffered from similar limitations in its observational nature we also found that implementation of a PE decision-support tool in the EMR across multiple EDs was associated with reduced CTA utilization and that diagnostic accuracy of CTA for suspected PE did not significantly improve with the decision-support tool. Given the large potential impact in reducing radiation exposure when applied at the system level we support the authors’ conclusion that implementation of EBM has demonstrated efficacy for reducing departmental CTA utilization.
